# Spatial Distribution and Conservation Prioritization of Medicinal Gymnosperms in China Using an Optimal Set‐Cover Approach

**DOI:** 10.1002/ece3.73613

**Published:** 2026-04-30

**Authors:** Lisi Hai, Xinyi Wang, Yuchang Yang, Rui Shu, Yanqin Xu, Guibing Lin, Lihua Dong, Xiaobo Zhang, Zhangjian Shan

**Affiliations:** ^1^ Jiangxi University of Chinese Medicine Nanchang China; ^2^ Dongguan Institute of Forestry Science Dongguan China; ^3^ State Key Laboratory for Quality Ensurance and Sustainable Use of Dao‐di Herbs, National Resource Center for Chinese Materia Medica China Academy of Chinese Medical Sciences Beijing China

**Keywords:** conservation gap, distribution pattern, medicinal gymnosperms, nature reserves

## Abstract

To elucidate the geographical distribution patterns and hotspots of medicinal gymnosperms in China, providing a scientific basis for formulating conservation strategies for this group, we compiled 17,999 occurrence records for 148 medicinal gymnosperm species native to China. Species were categorized into all, endemic, threatened, and nationally key protected medicinal gymnosperms groups. Distributions were analyzed across 943,100 × 100 km grid cells. Priority conservation areas were identified using an optimal algorithm based on an integer linear programming set‐cover formulation, which minimizes grids required to represent all species at least once, and compared with the Dobson algorithm. Conservation gaps were assessed by overlaying priority grids with national nature reserves and national parks. Medicinal gymnosperms showed a “more in the south, less in the north” distribution pattern, concentrated in mountainous areas and provincial borders. The distribution hotspots for all and endemic medicinal gymnosperms were in the Hengduan Mountains, while that for threatened and nationally key protected medicinal gymnosperms was in areas such as northern Guangxi. The optimal algorithm identified 41 priority conservation grids, mainly in areas like the border between Guizhou and Guangxi, outperforming the Dobson algorithm (which required 14% more grids on average). Overlay analysis revealed eight conservation gap grids, including high‐priority areas in the Hengduan Mountains. Current national reserves inadequately protect medicinal gymnosperms. Targeted conservation measures are needed based on identified gaps in this study. The optimal algorithm provides a resource‐efficient conservation tool. However, several limitations should be acknowledged. The use of 100 km grid cells, derived largely from county‐level records, may overestimate species' distribution areas and mask fine‐scale patterns. Uneven collection may also introduce spatial sampling biases. In addition, our analysis only considered national reserves, potentially overlooking local initiatives. Future work should incorporate higher‐resolution distribution data and sub‐national conservation designations to refine priority assessments.

## Graphical abstract

The study identified 41 priority conservation grids and 8 critical conservation gaps not covered by national reserves for medicinal gymnosperms. We proposed a practical, multi‐dimensional framework for their conservation.

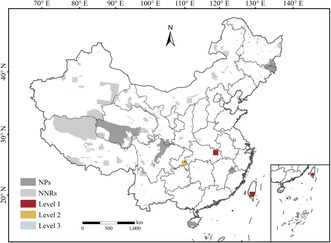

## 1 Introduction

Gymnosperms originated in the Middle Devonian approximately 385 million years ago (Ma), flourished during the Mesozoic, and have gradually declined since the Tertiary due to environmental changes, among other factors (Gerrienne et al. 2004; Crisp and Cook 2011; Wang and Ran 2014; Yang et al. 2017). Recent taxonomic studies indicate that extant gymnosperms worldwide comprise 3 classes, 5 subclasses, 8 orders, 13 families, 86 genera, and over 1000 species (Wang and Ran 2014; Yang et al. 2022). Despite the limited number of species, gymnosperms play crucial roles in ecosystems (Christenhusz et al. 2011). They are not only one of the five major seed plant lineages (Wang and Ran 2014; Xie et al. 2021), but also the main constructive species of forests worldwide. It is estimated that over 39% of the world's forests are composed of coniferous species, which play vital roles in the global carbon cycle and preventing soil erosion (Armenise et al. 2012; Wang and Ran 2014; Yang et al. 2017). Meanwhile, they possess economic value for ornamental use, food, construction, furniture, and industrial timber (Zonneveld 2011; Murray 2013; Wang and Ran 2014). Notably, some gymnosperms are also important sources of traditional and modern medicines, such as ephedrine from *Ephedra sinica* and paclitaxel from *Taxus chinensis* (Chen and Schmidt 1924; Schiff et al. 1979; Hu et al. 2021). Despite their significant ecological and economic importance, gymnosperms face multiple threats, with approximately 40% of species at risk of extinction (Brummitt et al. 2015; Fragnière et al. 2015; Forest et al. 2018).

Against the background of global warming, the vast majority of species are about to, are currently, or have already undergone range shifts in pursuit of suitable habitats. While some species may expand their ranges, the richness or distribution ranges of more species are shrinking, even facing extinction risks (Colwell et al. 2008; Chen et al. 2011; Gottfried et al. 2012; Li et al. 2021). Gymnosperms generally have long generation cycles and weak dispersal abilities, making them less adaptable to climate change (Aitken et al. 2008). Additionally, factors such as environmental pollution, invasive species, habitat destruction, and human disturbance significantly impact their survival (Chen et al. 2011). According to the latest IUCN Red List data, approximately 62.3% of cycad species and 33.3% of conifer species are threatened, with overexploitation and agricultural activities being the main threatening factors (https://www.iucnredlist.org/). However, beyond these widespread disturbances, gymnosperms are also constrained by narrow ecological niches, competition with angiosperms, and small population sizes (Schippmann et al. 2006; Larsen and Olsen 2007; Chen et al. 2016; Yang et al. 2017). Therefore, compared to other species, gymnosperm populations and their habitats are more vulnerable and urgently require focused conservation.

Clarifying the distribution pattern of biodiversity and priority conservation areas is crucial for formulating conservation strategies (Kreft and Jetz 2007; Li et al. 2021). Numerous studies have shown that the analysis of species' geographical distribution information helps to reveal their geographical distribution patterns (Cahyaningsih et al. 2021; Ye et al. 2023; Du et al. 2024). Du et al. (2024) analyzed the distribution information of 2855 medicinal plant species in tropical East Africa and found that areas with high medicinal plant diversity were mainly concentrated in northern and southern Rwanda, and the diversity pattern was highly similar to the overall vascular plant richness pattern. Ye et al. (2023), by comparing the spatial distribution information of native Chinese plants with *ex situ* conservation records, found conservation gaps for species in southwestern and northwestern China. After understanding distribution patterns, selecting appropriate areas for protection is another core issue. In practical conservation planning, due to limited resources such as manpower and funding, optimal resource allocation must be pursued. Common methods for solving conservation planning problems include the Dobson algorithm, Top 5% algorithm, etc., but these are not optimal choices under resource constraints (Beyer et al. 2016). The optimal algorithm, by minimizing or maximizing an objective function (a mathematical equation describing the relationship between actions and outcomes) while satisfying constraints, can yield optimal solutions, achieving maximum conservation efficiency with minimal resource input (Beyer et al. 2016).

China harbors the richest gymnosperm resources in the world, and its southwestern region is recognized as one of the five global hotspots of gymnosperm diversity (Contreras‐Medina et al. 2001; Barthlott et al. 2007; Li et al. 2009). Chinese gymnosperms account for approximately 22.2% of the global total, with endemic species comprising 42.8% (Yang 2015; Zhou et al. 2021). Previous studies have examined Chinese gymnosperms from several perspectives. For example, Li et al. (2021) analyzed spatial patterns and conservation priority regions for gymnosperms in China as a whole, whereas Xie et al. (2021) focused specifically on threatened gymnosperms. In addition, studies relevant to medicinal gymnosperms have mainly focused on broader plant groups rather than on medicinal gymnosperms specifically. For example, Chi et al. (2017) explored the distribution patterns and conservation priorities of threatened medicinal plants in China based on county‐level records. More recently, Pan et al. (2025) evaluated conservation gaps for nationally key protected wild plants. However, a comprehensive assessment focused specifically on medicinal gymnosperms in China remains lacking. This gap is important because medicinal gymnosperms represent a distinct conservation‐relevant subgroup whose extinction would imply not only biodiversity loss but also the loss of potentially valuable medicinal resources (Schippmann et al. 2006; Chen et al. 2016).

Accordingly, this study focuses on medicinal gymnosperms in China and integrates multiple conservation dimensions—including species richness, endemism, threat status, and national protection status—within an optimization‐based set‐cover framework to identify priority conservation areas and conservation gaps. Specifically, we ask: (1) do different conservation dimensions of medicinal gymnosperms exhibit similar spatial hotspot patterns? (2) can an optimal set‐cover algorithm identify priority conservation areas more efficiently than the commonly used Dobson algorithm? and (3) do priority conservation areas identified by the optimization approach fall outside the current network of national nature reserves and national parks, thereby revealing conservation gaps? By addressing these questions, we aim to provide a more targeted basis for conservation planning for medicinal gymnosperms in China and a transferable framework for other conservation‐relevant plant groups.

## 2 Methods and Materials

### 2.1 Data Collection

#### 2.1.1 Compilation of the List of Medicinal Gymnosperm Species in China

Based on data from the Fourth National Survey of Chinese Materia Medica Resources (Huang et al. 2024), an initial list of 184 medicinal gymnosperm species recorded in China was compiled (Table S1). In this study, medicinal gymnosperms were defined as gymnosperm species documented as having medicinal uses in this survey database. Species were included if any plant part was reported as medicinal, regardless of whether the species was widely used in formal traditional Chinese medicine or primarily recorded in local or folk medicinal practices.

Taxonomic names were standardized primarily following *Flora of China* (Wu et al. 1994–2013). When taxa were not included in this reference (e.g., *Larix potaninii* var. *macrocarpa* and *Pinus kesiya* var. *langbianensis*), their accepted names were verified using the Plants of the World Online (POWO) database (https://powo.science.kew.org/). Non‐native medicinal species (e.g., cultivated or introduced taxa) were excluded. Synonyms were merged under the currently accepted species names, and infraspecific taxa were retained as recorded in the original dataset. After taxonomic standardization and data cleaning, the final dataset comprised 148 native Chinese medicinal gymnosperm species belonging to 10 families and 35 genera (Table S2).

Based on this dataset, three additional subsets were derived according to different conservation dimensions. First, endemic medicinal gymnosperms were identified by tracing species origins using *Flora of China* and the POWO, resulting in 61 species belonging to 9 families and 22 genera (Table S3). Second, threatened medicinal gymnosperms were identified based on the China Biodiversity Red List—Higher Plants (https://www.mee.gov.cn/xxgk2018/xxgk/xxgk01/202305/t20230522_1030745.html), which applies the IUCN Red List Categories and Criteria at the national scale; this subset included 50 species from 9 families and 28 genera (Table S4). Third, nationally key protected medicinal gymnosperms were extracted using the species‐level checklist compiled by Pan et al. (2025), which resolves higher‐level taxonomic groups listed in the List of National Key Protected Wild Plants into species‐level records. This subset comprised 43 species belonging to 9 families and 23 genera (Table S5).

#### 2.1.2 Collection and Processing of Species Distribution Data for Chinese Medicinal Gymnosperms

The distribution data for medicinal gymnosperms in this study were derived primarily from the Fourth National Survey of Chinese Materia Medica Resources (Huang et al. 2024). Meanwhile, species distribution data from Shan et al. (2022) and Pan et al. (2025) were obtained, which extensively collected county‐level geographical distribution records from national and local floras, checklists, herbaria, and published literature.

Preliminary, 17,999 county‐level occurrences for 148 Chinese medicinal gymnosperms were compiled (Table S6). Some of the original records in these datasets were standardized from broader geographic descriptions (e.g., mountain ranges or regional locations reported in floristic literature). Subsequently, considering the spatial precision of the original occurrence records is heterogeneous and often lacks fine‐scale geographic coordinates, species occurrences were projected onto a standardized 100 × 100 km grid. Then, we selected the grid‐cell occurrence data with two steps: (1) Records were removed if they located in grid cells where the actual area in border and coastline regions is less than 50% of the grid area, as they would affect the actual analysis. (2) Only one record was retained if multiple county‐level distribution data of the same species fall within the same grid. This approach helps harmonize spatial uncertainty among records and provides consistent planning units for national‐scale spatial analyses. Ultimately, we obtained 6993 grid‐cell occurrence records for Chinese medicinal gymnosperms, where each record represents the presence of a species within a grid cell (Table S7). From this total, we extracted records belonging to species in three conservation categories: 2922 records for endemic species, 1355 records for threatened species, and 1566 records for nationally key protected species (Table S7).

It is important to note that these categories are not mutually exclusive; a single species—and thus its distribution records—may fall into multiple categories (e.g., a species can be both endemic and threatened). *Flora of China* records *Cedrus deodara* as distributed in southwestern Tibet (Wu et al. 1994–2013), but no reliable specimen records were found; therefore, it was excluded from subsequent distribution and conservation analyses.

#### 2.1.3 Acquisition of Geographical Data for National Nature Reserves in China

The layer of Chinese national nature reserves was obtained from the Resource and Environmental Science Data Platform (https://www.resdc.cn/data.aspx?DATAID=272), which provides boundaries for 477 national nature reserves in China up to 2018. The geographical distribution layers for five national parks were obtained from Pan et al. (2025).

### 2.2 Analytical Methods

#### 2.2.1 Statistical Analysis of Diversity of Chinese Medicinal Gymnosperms

To understand the diversity of Chinese medicinal gymnosperms, this study summarized them from four perspectives: (1) Endemism of Chinese medicinal gymnosperms; (2) Threat status (Critically Endangered, Endangered, Vulnerable) of Chinese medicinal gymnosperms; (3) National key protection status of Chinese medicinal gymnosperms; (4) Classification of medicinal parts of Chinese medicinal gymnosperms. For medicinal parts, records were made and classified based on medical classics, published monographs, and literature (Huang et al. 2024), with the classification standard slightly adjusted from Shan et al. (2023) into three categories: Category I includes parts whose use easily leads to plant death (whole plant, roots, heartwood); Category II includes parts harmful but not fatal (branches/leaves, resin, bark); Category III includes parts causing minimal harm (flowers, fruits, seeds) (Table S8). Some species are recorded as having multiple medicinal parts belonging to different categories. In Table S8, species with multiple medicinal parts were counted in each relevant category.

Statistical analysis and visualization of diversity patterns were conducted for these four aspects.

#### 2.2.2 Statistical Analysis of Relationships Between Threat Status, Medicinal Parts, and Endemism

To further explore factors associated with the conservation status of medicinal gymnosperms, we examined whether threat levels are related to the type of medicinal parts used and whether endemic species are disproportionately represented among threatened taxa. Endemic status was determined based on Table S3, which lists species native exclusively to China. Threat status for each species was obtained from the IUCN Red List categories (CR, EN, VU) compiled in Table S4. Medicinal part categories were defined as described above and summarized in Table S8. For species recorded with multiple medicinal parts spanning different impact categories, each species was assigned to the category representing the highest potential impact (I > II > III). This conservative approach assumes that the presence of any fatal‐part use (e.g., whole plant or roots) introduces the potential for destructive harvesting, even if less harmful parts are also used. Species using only Categories II and/or III were grouped as “non‐fatal parts” for statistical comparisons. Each species was counted only once to avoid double counting. Contingency tables were constructed for (i) threat status (threatened vs. non‐threatened) × medicinal part category (fatal vs. non‐fatal) and (ii) threat status × endemism (endemic vs. non‐endemic). Chi‐square tests of independence were used to evaluate statistical associations, with a significance level of α = 0.05. All analyses were conducted in R (version 4.5.2).

#### 2.2.3 Visualization of Distribution Patterns of Chinese Medicinal Gymnosperms

First, the map of China was gridded using ArcGIS v10.8.2 (ESRI Inc., Redlands, CA, USA; http://www.esri.com/software/arcgis). To eliminate the influence of administrative area size differences, China was divided into 1172 equal‐area grids of 100 km × 100 km (Lu et al. 2018). To mitigate the effects of grid incompleteness in border and coastal areas, grids with actual areas less than 50% of the grid area were removed from subsequent analysis, resulting in 943 final grids (Table S9).

Second, distribution pattern analysis was performed. The grid‐cell occurrence records of the four categories of medicinal gymnosperms (all, endemic, threatened, nationally key protected) were projected onto the grid layer. Species richness for each grid cell was calculated as the number of species recorded within that cell (Crisp et al. 2001; Zhang et al. 2015). Richness maps were generated and visualized in ArcGIS v10.8.2 (ESRI Inc., Redlands, CA, USA; http://www.esri.com/software/arcgis).

#### 2.2.4 Identification of Priority Conservation Areas and Conservation Gaps for Chinese Medicinal Gymnosperms

To concentrate resources and lower the cost, using the least number of grid cells to cover all target species is a common strategy in conservation. Dobson et al. (1997) solved such problems intuitively by in each step selecting one grid cell which covered the most previously uncovered target species; however, this solution yields merely a relatively small set of grid cells, with its number not guaranteed to be minimum. Mathematically, finding the best solution of this minimum set cover problem is believed to be non‐deterministic polynomial hard, but in the framework of integer linear programming theory, there have been complicated but efficient solvers applicable (Underhill 1994; Ando et al. 1998; Moore et al. 2003). For such problems, Yang et al. (2022) developed a Julia package SetCover.jl providing heuristic solution from Dobson et al. (1997)'s algorithm and optimal solution from modern integer linear problem solvers.

Following the framework of minimum set cover problem and the SetCover.jl package routines, considering a certain category of medicinal gymnosperm species and their distribution in 100 km × 100 km grid cells, we prepared the lists of grid cells occupied for every target species. Using these lists, we applied the SetCover.jl package to compute a heuristic grid cell list (Dobson et al. 1997) and an optimal grid cell list (Ando et al. 1998) respectively using fewer and fewest grid cells to cover all these medicinal gymnosperm species. The process was executed for the four categories of medicinal gymnosperm species (all, endemic, threatened, nationally key protected). We defined priority conservation area in this study comprising the grid cells in the four optimal grid cell lists corresponding to the four categories. The grid cells present on exactly *k* optimal lists defined the Level 5‐*k* priority conservation area, *k* = 1,2,3,4.

Establishing nature reserves is a primary strategy for biodiversity conservation worldwide (Xu et al. 2019). Since 1956, China has established 2750 nature reserves of various types and administrative levels, including 477 national nature reserves and five national parks (https://www.resdc.cn/data.aspx?DATAID=272). In general, national‐level reserves receive greater financial and administrative support and therefore tend to achieve stronger conservation outcomes, whereas many sub‐national reserves face limitations in funding, staffing, and management capacity (Xu et al. 2017). To ensure consistent evaluation of conservation effectiveness at the national scale, this study focuses on national nature reserves and national parks. Spatial layers of these protected areas were overlaid with the four levels of priority conservation grids to assess protection coverage. Grid cells lacking any overlap with national‐level protected areas were defined as conservation gap grids (Pan et al. 2025).

## 3 Results

### 3.1 Diversity of Medicinal Gymnosperms

The results show that China has 148 species of medicinal gymnosperms, belonging to 5 subclasses, 10 families, and 35 genera, accounting for approximately 47% of the total gymnosperm species in China. The Pinidae subclass had the highest number of species, reaching 67 (Table S2).

**FIGURE 1:**
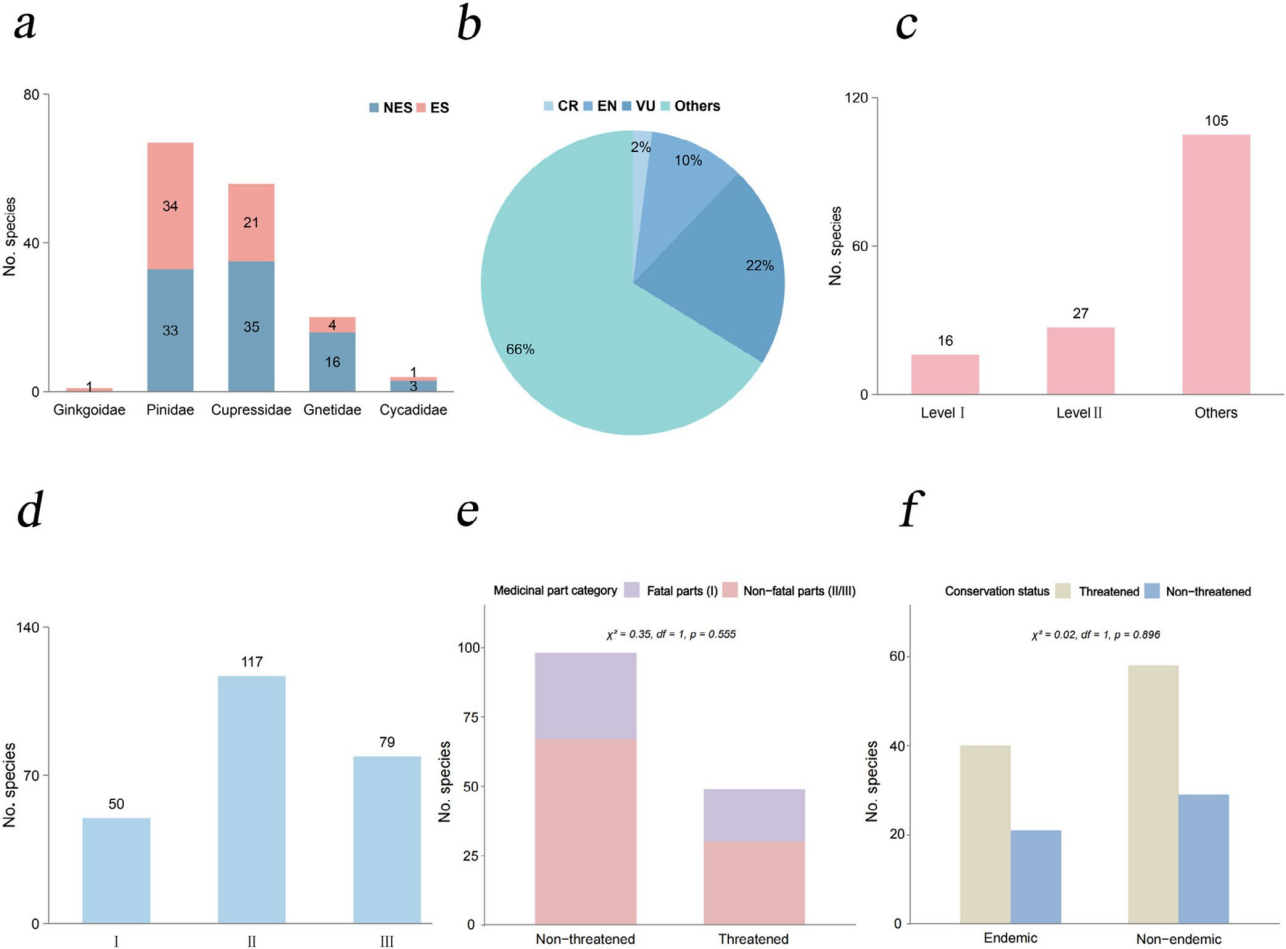
Diversity of medicinal gymnosperms in China. (a) Endemism of Chinese medicinal gymnosperms (NES: Non‐endemic species; ES: Endemic species); (b) Threat status of Chinese medicinal gymnosperms; (c) National key protection status of Chinese medicinal gymnosperms; (d) Classification of medicinal parts of Chinese medicinal gymnosperms (I: Whole plant, roots, heartwood; II: Branches/leaves, resin, bark; III: Flowers, fruits, seeds, see Additional file 1: Table S8 for details). (e) Comparison of medicinal part categories (fatal parts vs. non‐fatal parts) between threatened and non‐threatened species. Fatal parts include whole plant, roots, and heartwood (Category I); non‐fatal parts include branches/leaves, resin, bark (Category II) and flowers, fruits, seeds (Category III). Chi‐square test showed no significant association (χ^2^ = 0.35, df = 1, *p* = 0.555). (f) Comparison of threat status between endemic and non‐endemic species. No significant difference was detected (χ^2^ = 0.02, df = 1, *p* = 0.896).

Endemic Chinese medicinal gymnosperms are distributed across all 5 gymnosperm subclasses (Table S3). The Ginkgoidae subclass, with only *Ginkgo biloba* , has an endemism rate of 100%; the Pinidae subclass has the next highest endemism rate at about 50.7%, and its endemic species account for about 41.2% of the total Chinese medicinal gymnosperms (Figure 1a, Table S3).

Among all Chinese medicinal gymnosperms, according to the IUCN Red List categories, there are 3 Critically Endangered (CR), 15 Endangered (EN), and 32 Vulnerable (VU) species, accounting for 2%, 10%, and 22% of all Chinese medicinal gymnosperms, respectively (Figure 1b, Table S4).

Among all Chinese medicinal gymnosperms, 16 species are under National Level I protection and 27 under National Level II protection, with nationally protected species accounting for about 29% of the total (Figure 1c, Table S5).

For medicinal plants, the medicinal parts are not single; multiple parts of a plant can be used medicinally (Coley et al. 2003). The classification of medicinal parts of gymnosperms in this study showed that the most frequently used parts were those causing some harm but not fatal to the plant (branches/leaves, resin, bark); followed by parts causing minimal harm (flowers, fruits, seeds); while the use of parts that may lead to plant death (whole plant, roots, heartwood) accounted for about one‐third of all medicinal gymnosperms (Figure 1d).

### 3.2 Relationships Between Threat Status, Medicinal Parts, and Endemism

Among the 148 medicinal gymnosperms, 68 species (45.9%) are used for a single medicinal part category, while 80 species (54.1%) are used for multiple categories. Of the 80 multi‐category species, 42 involve both fatal (Category I) and non‐fatal parts (Categories II/III). For the chi‐square analysis, these 42 species were classified as fatal‐part users, consistent with our conservative approach. Among the 50 threatened medicinal gymnosperm species in China (Table S4), 19 species (38%) are harvested for fatal parts (Category I). In comparison, among the 98 non‐threatened medicinal gymnosperms, 31 species (31.6%) involve fatal parts. A chisquare test revealed no significant association between threat status and the use of fatal parts (*χ* ^2^ = 0.‐35, df = 1, *p* = 0.555; Figure 1e).

Of the 61 endemic medicinal gymnosperm species (Table S3), 21 (34.4%) are classified as threatened. Among the 87 nonendemic species, 29 (33.3%) are threatened. This difference was not statistically significant (*χ* ^2^ = 0.02, df = 1, *p* = 0.896; −Figure 1f).

### 3.3 Distribution Patterns and Hotspot Areas of the Four Categories of Medicinal Gymnosperms

**FIGURE 2:**
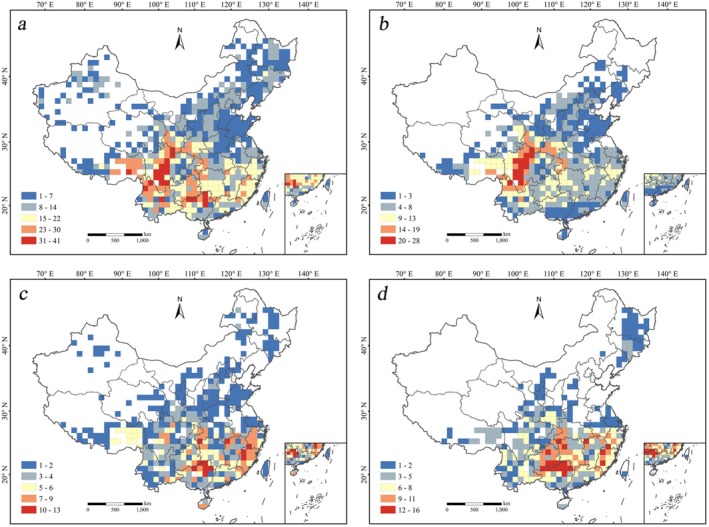
Distribution patterns of the four categories of Chinese medicinal gymnosperms. (a) Distribution pattern of all Chinese medicinal gymnosperms; (b) Distribution pattern of endemic Chinese medicinal gymnosperms; (c) Distribution pattern of threatened Chinese medicinal gymnosperms; (d) Distribution pattern of nationally key protected Chinese medicinal gymnosperms.

The distributions of all, threatened, endemic, and nationally key protected medicinal gymnosperms in China all exhibited a spatial pattern of “high in the south, low in the north.” Species richness was higher in mountainous areas and sparse and scattered in northern plateaus, plains, and basins (Figure 2).

Medicinal gymnosperms are widely distributed in China, covering 578 out of 943 grids (Table S9). Among all medicinal gymnosperms, 51 species have distribution ranges exceeding 50 grids, with *Platycladus orientalis* having the widest distribution (246 grids), followed by *Cunninghamia lanceolata* (223 grids), *Cephalotaxus fortunei* (219 grids), and *Pinus massoniana* (204 grids) (Table S10). The core distribution area for medicinal gymnosperms is the Hengduan Mountains region in southwestern China, with scattered hotspot grids also found in northern Guangxi, the eastern section of the Nyainqêntanglha Mountains, and central Yunnan (Figure 2a). The grid with the highest species richness in these areas contains about 41 medicinal gymnosperm species (28% of the total) (Table S9).

Endemic Chinese medicinal gymnosperms are distributed in 460 out of 943 grids (Table S9). *Pinus massoniana* has the widest distribution (204 grids), followed by *Cupressus funebris* (186 grids), *Cephalotaxus sinensis*, and *Juniperus formosana* (both 184 grids) (Table S10). About 57% of endemic medicinal gymnosperm species have distribution ranges of less than 50 grids, with 11 species having ranges of less than 10 grids. The species diversity hotspots for endemic medicinal gymnosperms are concentrated in the Hengduan Mountains region, where each grid contains an average of about 24 species (39% of the total endemic medicinal gymnosperms); additionally, species richness is also high in areas like the Qinling Mountains, Daba Mountains, and Wuling Mountains (Figure 2b).

Threatened Chinese medicinal gymnosperms are distributed in 416 out of 943 grids. *Taxus wallichiana* has the widest distribution (178 grids) (Table S9), but 80% of threatened medicinal gymnosperm species have distribution ranges of less than 50 grids (Table S10). The distribution hotspots for threatened medicinal gymnosperms are mainly located in northern Guangxi and the Wuyi Mountains, with scattered diversity hotspots also in the Wuling Mountains, Mufu Mountains, and Jiuling Mountains; species richness is high in areas like Tianmu Mountain, Xianxialing, and Yandang Mountain, and relatively concentrated areas of higher richness also exist in the Himalayas and the eastern Nyainqêntanglha Mountains (Figure 2c).

Nationally key protected Chinese medicinal gymnosperms are distributed in 329 out of 943 grids (Table S9). *Taxus wallichiana* has the widest distribution range. About 70% of nationally key protected medicinal gymnosperms have distribution ranges of less than 50 grids, with 9 species having ranges of less than 10 grids (Table S10). The distribution pattern shows that species diversity hotspots are mainly located in northern Guangxi and southern Guizhou, with scattered hotspots also in the Dalou Mountains, Wuling Mountains, Mufu Mountains, Jiuling Mountains, Wuyi Mountains, and Daiyun Mountains. Grids in these areas contain an average of about 13 nationally key protected species (30% of the total). Furthermore, species richness is high in the eastern Daba Mountains, Xuefeng Mountains, southern Luoxiao Mountains, Tianmu Mountain, and Xianxialing (Figure 2d).

### 3.4 Priority Conservation Area Grids for the Four Categories of Medicinal Gymnosperms

**FIGURE 3:**
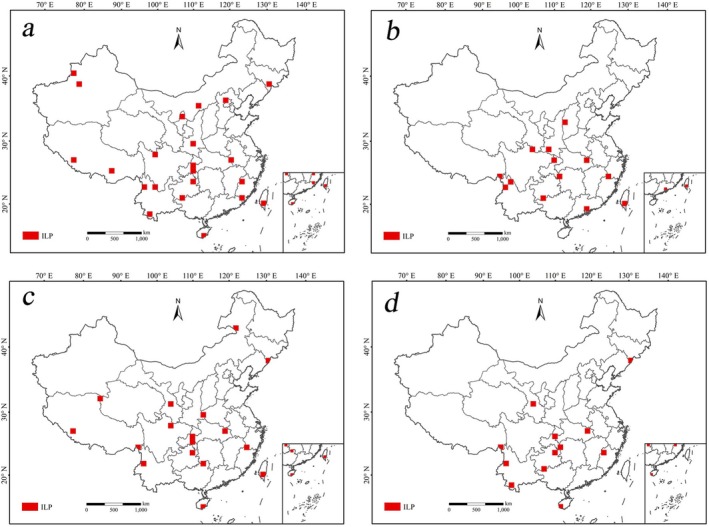
Priority conservation areas for the four categories of Chinese medicinal gymnosperms. (a) Priority conservation areas for all Chinese medicinal gymnosperms; (b) Priority conservation areas for endemic Chinese medicinal gymnosperms; (c) Priority conservation areas for threatened Chinese medicinal gymnosperms; (d) Priority conservation areas for nationally key protected medicinal gymnosperms.

This study used the optimal algorithm to calculate priority conservation areas for medicinal gymnosperms. The results are as follows: priority conservation areas for all Chinese medicinal gymnosperms comprise 22 grids (Figure 3a; Table S11); for endemic medicinal gymnosperms, 13 grids (Figure 3b; Table S11); for threatened medicinal gymnosperms, 17 grids (Figure 3c; Table S11); for nationally key protected medicinal gymnosperms, 12 grids (Figure 3d; Table S11).

The priority conservation areas for the four categories total 41 grids, covering approximately 4.3% of China's land area and encompassing 143 species (Table S9, Table S11). They are mainly distributed in mountainous areas and provincial border regions. Except for *Cedrus deodara* (excluded due to lack of distribution records) and *Pinus sylvestris* var. *sylvestriformis*, *Juniperus sabina* var. *davurica*, *Cephalotaxus griffithii*, and *Ephedra glauca* (deleted because they were located in grids with area < 50%), the identified priority conservation areas cover all Chinese medicinal gymnosperms. Among these 41 grids, 35 grids contain more than 10 medicinal gymnosperm species each, with the most species‐rich grid containing 39 species (Table S9, Table S11).

To validate the effectiveness of the optimal algorithm, the Dobson algorithm was also used to calculate priority conservation areas for the four categories as a control (Dobson et al. 1997). The results showed that, under the premise of protecting the same number of species, the Dobson algorithm identified 26 grids for all medicinal gymnosperms, 15 for endemic, 18 for threatened, and 14 for nationally key protected medicinal gymnosperms, which are generally more than the results from the optimal algorithm (Table S12).

**FIGURE 4:**
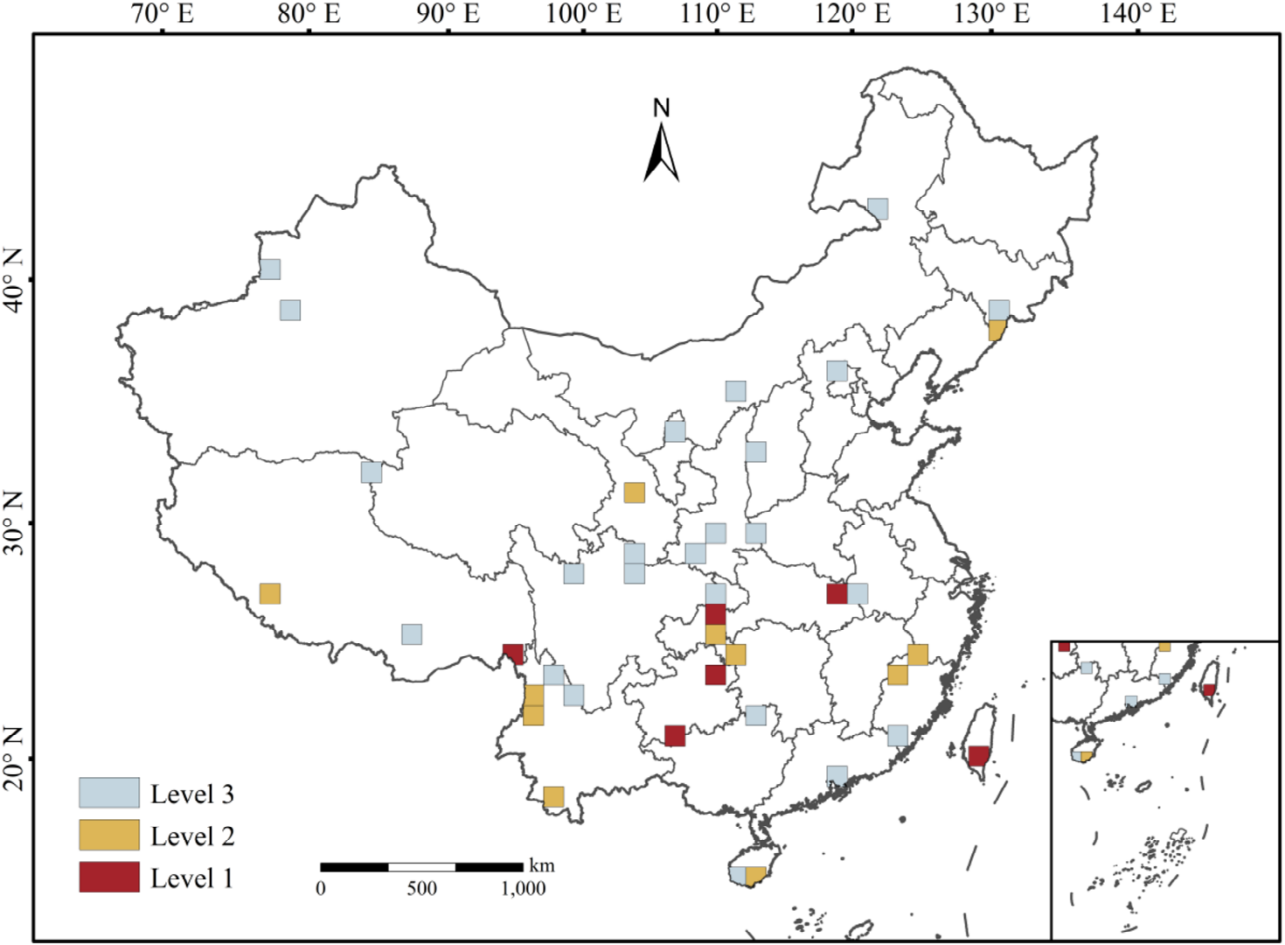
Priority conservation levels for Chinese medicinal gymnosperms.

To enhance the operability and efficiency of conservation practice, the 41 grids were graded according to priority conservation levels (Figure 4), focusing priority on grids identified by multiple lists, as species within them are considered more ecologically significant. Since no grid was identified as a priority by all four lists simultaneously, grids identified by three lists were defined as Level 1 priority areas, and those grids identified by two lists and one list were defined as Level 2 and Level 3, respectively. The specific grading results are: Level 1 priority areas comprise 6 grids, mainly located in the southern section of the Shubola Mountains, the border between Guizhou and Guangxi, the Tongren area, southern Taiwan Province, the border between Huanggang City and Wuhan City, and the border between Hubei and Chongqing; Level 2 priority areas comprise 11 grids, mainly distributed in southern Hainan Province, southern Yunnan Province, Gaoligong Mountains, Wuling Mountains, Dalou Mountains, Wuyi Mountains, Gangdise Mountains, central and southern Gansu, and the southern section of the Changbai Mountains; Level 3 priority areas comprise 24 grids, mainly located in southern Hainan Province, central and southern Guangdong Province, Daiyun Mountains, the border between Guangxi and Hunan, etc. (Table S12).

### 3.5 Conservation Gaps for the Four Categories of Medicinal Gymnosperms

**FIGURE 5:**
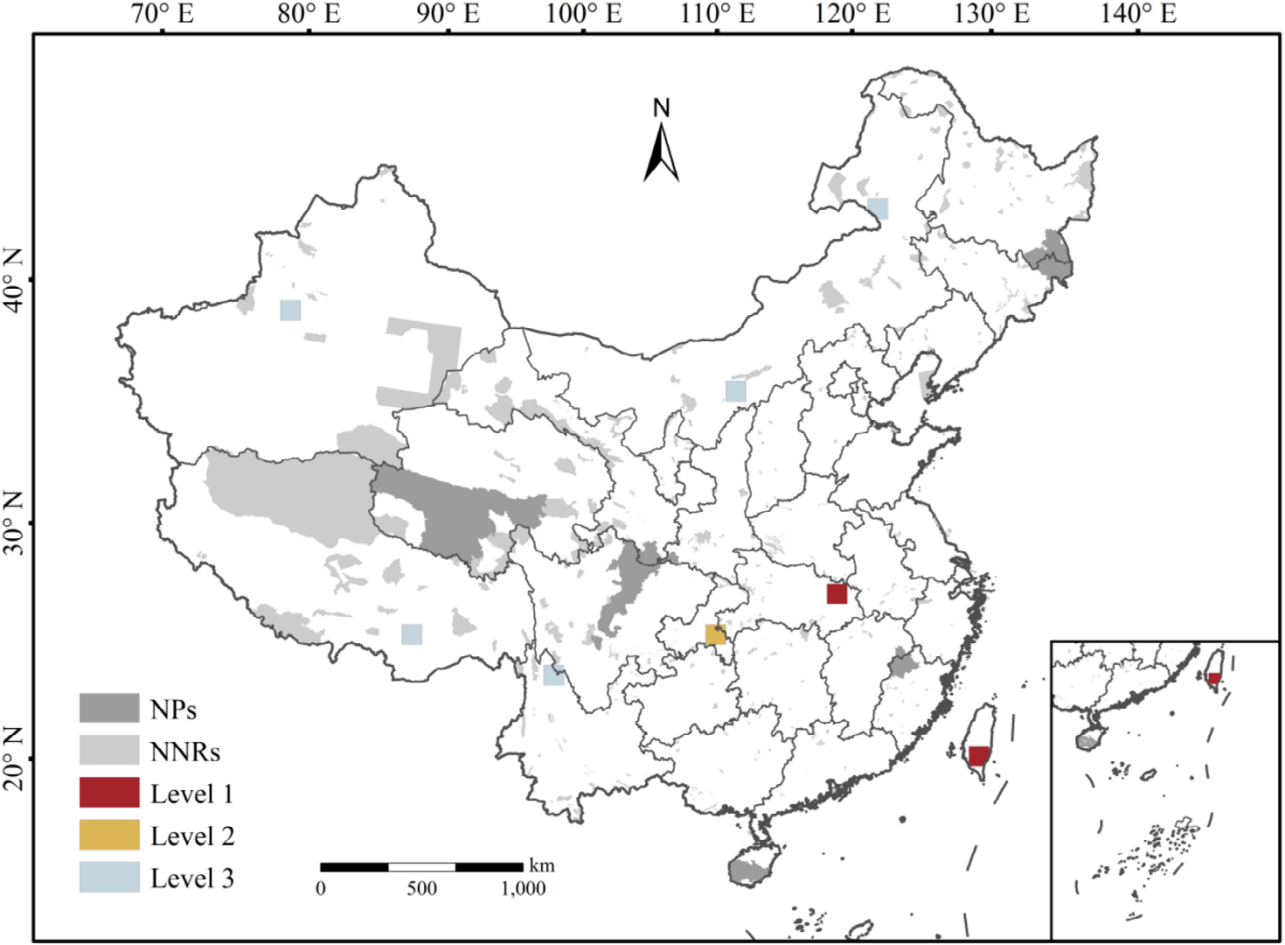
Conservation gap levels for Chinese medicinal gymnosperms.

Overlaying the obtained national nature reserve and national park layers with the 41 priority conservation grids showed that 33 priority grids overlapped with existing reserve boundaries (considered protected areas), while 8 grids were not covered by any existing reserve, thus identified as conservation gaps (Figure 5; Table S13). Among Level 1 priority grids, 2 grids lack national nature reserves or national parks, located in southern Taiwan Province and the border between Anhui and Hubei provinces; among Level 2 priority grids, 1 grid lacks protection, located in the northern section of the Dalou Mountains; among Level 3 priority grids, 5 grids lack protection, located in northern Yunnan Province, the eastern Himalayas, northeastern Aksu Prefecture in Xinjiang, northeastern Ordos City in Inner Mongolia, and northern Hulunbuir City (Figure 5; Table S13).

These conservation gaps all have high species conservation value. The grid with the highest species richness (located within Daocheng County and Muli Tibetan Autonomous County, Sichuan Province) contains 37 species of medicinal gymnosperms; another grid at the border between Jinzhai County, Anhui Province and Luotian County, Hubei Province, contains 22 species of threatened, endemic, and nationally key protected medicinal gymnosperms combined, making it highly significant for maintaining medicinal gymnosperm diversity (Figure 5; Table S13).

## 4 Discussion

### 4.1 Species and Medicinal Part Diversity of Chinese Medicinal Gymnosperms

In long‐term medical practice, the Chinese people have accumulated valuable experience in using gymnosperms for disease prevention and treatment. Ancient Chinese medical works such as “Shennong Ben Cao Jing”, “Compendium of Materia Medica” (Bencao Gangmu), and “An Illustrated Book of Plants” (Zhiwu Mingshi Tukao) already recorded the medicinal values of gymnosperms like Ginkgo, Podocarpus, and Ephedra. This study systematically compiled a list of 148 native Chinese medicinal gymnosperm species belonging to 10 families and 35 genera based on the Fourth National Survey of Chinese Materia Medica Resources (Table S2). At the family level, Chinese medicinal gymnosperms are distributed across all 10 families, with Pinaceae having the highest number; at the genus level, the genus with the most medicinal gymnosperms is *Pinus*, accounting for about 48% of the species in that genus. This might be because Pinaceae and *Pinus* inherently have high species numbers, but *Pinus* is not the genus with the highest medicinal proportion; some genera with fewer species even reach 100% medicinal use proportion.

The medicinal value of gymnosperms stems from secondary metabolites produced during their growth and metabolism, which accumulate to varying degrees in different medicinal parts (e.g., roots, stems, leaves, flowers, fruits, seeds) (Min et al. 2023; McClune et al. 2025). This study found that for most Chinese medicinal gymnosperm species, use focuses on parts not fatal to the plant (e.g., branches/leaves, resin, bark). However, since the use of medicinal parts is not exclusive, one‐third still utilize parts that lead to plant death (Figure 1d; Table S8). It is particularly important to note that about 83% of medicinal gymnosperms are coniferous trees, which have long generation cycles and weak population recovery capabilities. Destructive harvesting can easily lead to sharp population declines, habitat fragmentation, or even extinction (Aitken et al. 2008; Murray 2013; Wang and Ran 2014). Contrary to expectations, our analysis revealed no significant association between the harvesting of fatal parts (Category I) and the threat status of Chinese medicinal gymnosperms (Figure 1e). Although destructive harvesting practices—such as uprooting whole plants or collecting roots and heartwood—have been widely implicated in the overexploitation of medicinal species (Schippmann et al. 2006; Larsen and Olsen 2007), the absence of a statistical relationship in our dataset may be explained by several factors. Many species harvested for fatal parts, such as *Pinus massoniana* and *Cunninghamia lanceolata* , are widespread and common (Table S10), rendering them less susceptible to extinction despite intensive use. Conversely, several threatened species in our study are primarily harvested for non‐fatal parts (e.g., foliage or bark of *Taxus wallichiana*), suggesting that other drivers—such as habitat loss, intense competition with angiosperms, narrow ecological niches, or inherently small population sizes—may be more critical determinants of threat status (Xiong et al. 2006; Brodribb et al. 2012; Yang et al. 2017). Similarly, we found no significant difference in the proportion of threatened species between endemic and non‐endemic taxa (Figure 1f). While endemism is often linked to elevated extinction risk due to restricted ranges and specialized habitats, many Chinese endemic medicinal gymnosperms, including *Cupressus funebris* and *Pinus tabuliformis* , maintain relatively large populations and broad distributions (Table S10). In contrast, non‐endemic species face severe threats across their limited ranges due to habitat degradation and historical overexploitation. These results underscore that threat status in medicinal gymnosperms is shaped by a complex interplay of factors, and neither the type of plant part used nor endemism alone serves as a reliable proxy for extinction risk.

It should also be acknowledged that ethnopharmacological knowledge and use patterns are not static; the parts of a species used medicinally may shift over time in response to changing commercial demand, cultural transmission, or resource scarcity (Chen et al. 2016). Our classification, based on current literature and pharmacopeias, represents a snapshot of documented uses and may not fully capture historical or future variations in harvesting practices. This temporal uncertainty could influence the perceived relationship between medicinal part usage and threat status, particularly for species that have experienced recent shifts in utilization pressure.

### 4.2 Distribution Patterns of Four Categories of Medicinal Gymnosperms

Based on the compiled lists of all, endemic, threatened, and nationally key protected medicinal gymnosperms in China and the established county‐level species distribution database, this study revealed the distribution patterns and their similarities and differences for the four categories. We found that all Chinese medicinal gymnosperms are mainly distributed in mountainous areas of southwestern and southern China, particularly centered in the Hengduan Mountains region (Figure 2a), which is largely consistent with previous studies on gymnosperm distribution patterns (Contreras‐Medina et al. 2001; Lv et al. 2018; Li et al. 2021). This pattern may stem from the following reasons. First, habitat adaptation dominates. Most medicinal gymnosperms (e.g., Podocarpaceae, Taxaceae, Cycadaceae, Gnetaceae) prefer warm and humid environments, thus are mainly distributed in the south (Pittermann et al. 2012; Leslie et al. 2012; Lv et al. 2018). Second, advantage of habitat heterogeneity. The high habitat heterogeneity in the Hengduan Mountains region provides more ecological niches for species coexistence, thus supporting more species (Currie 1991; Stein et al. 2014). Furthermore, mountains can act as refugia for species during rapid environmental and climate changes, preserving more species (Cun and Wang 2010; Chen et al. 2011; Sandel et al. 2011). Especially since the Tertiary period, the stable climate and diverse habitats of the Hengduan Mountains region have protected many ancient medicinal gymnosperms (Currie 1991; Qian and Ricklefs 2000; Qiu et al. 2017). Third, environmental drivers of medicinal compound accumulation. The synthesis and accumulation of secondary metabolites in medicinal plants are influenced by both genetic and environmental factors (Ramakrishna and Ravishankar 2011; Pant et al. 2021). The complex and diverse microhabitats in mountainous areas provide favorable conditions for the accumulation of active ingredients in medicinal gymnosperms (Körner and Spehn 2002; Tang et al. 2006).

Our finding that all medicinal gymnosperms are concentrated in the Hengduan Mountains region (Figure 2a) confirms the well‐established role of this region as a major biodiversity center for gymnosperms in China (Contreras‐Medina et al. 2001; Li et al. 2021). Similarly, the concentration of endemic species in the Hengduan Mountains (Figure 2b) aligns with previous phylogeographic studies identifying this area as a center of endemism and species differentiation (Cun and Wang 2010). In contrast, the distribution patterns of threatened and nationally key protected medicinal gymnosperms reveal a previously unreported pattern: their hotspots in northern Guangxi and the Wuyi Mountains (Figure 2c,d) are spatially distinct from the endemic species hotspot. This mismatch—particularly the limited spatial overlap between endemic and nationally protected species hotspots—highlights a potential gap in current conservation prioritization for medicinal gymnosperms.

Further analysis showed that the distribution patterns of all and endemic Chinese medicinal gymnosperms are highly similar, with diversity hotspots for both located in the Hengduan Mountains region (Figure 2a,b). Throughout Earth's history, major geological events and climate change processes have driven changes in species distribution ranges (migration), evolution of genetic structure (differentiation), and even complete species disappearance (extinction) through strong selective pressures (Qi et al. 2012; Bai et al. 2016). Complex mountain environments can promote species differentiation through geographical isolation (Cun and Wang 2010; Pittermann et al. 2012; Qiu et al. 2017). In this study, the Hengduan Mountains cover 62% of endemic medicinal gymnosperm species, and the distribution pattern of endemic Chinese medicinal gymnosperms differs from the other three categories, with its species diversity hotspots highly concentrated in the Hengduan Mountains region. This supports the view that the Hengduan Mountains are a center of species diversity and differentiation (Cun and Wang 2010).

The distribution patterns of nationally key protected and threatened Chinese medicinal gymnosperms also show high similarity, with distribution centers mainly located in northern Guangxi and the Fujian‐Zhejiang mountainous area (Figure 2c,d). This is closely related to the selection criteria for nationally key protected species—such species are often endangered groups with narrow distributions and small populations (Lu et al. 2021). Notably, the distribution patterns of nationally key protected and endemic Chinese medicinal gymnosperms differ significantly, with their species diversity hotspots almost non‐overlapping. This suggests that the current List of National Key Protected Wild Plants in China may be insufficient in protecting endemic medicinal gymnosperms. Endemic species, especially endemic medicinal plants, may contain unknown special chemical components with the potential to become key lead compounds for new drug development, which could be lost if the species become extinct (Baker et al. 2007). In light of this, it is recommended to strengthen attention to endemic medicinal gymnosperms in future updates of the List of National Key Protected Wild Plants in China.

The richness hotspots for threatened Chinese medicinal gymnosperms are mainly located in northern Guangxi and the Wuyi Mountains (Figure 2c). Also based on the IUCN Red List, Xie et al. (2021) found that the area with the highest species richness of threatened gymnosperms in China was located on the Western Sichuan Plateau. This difference may arise from the different datasets used: this study focused on threatened medicinal gymnosperms, while the latter covered all threatened gymnosperms. Their distribution patterns are similar, but the specific diversity hotspot areas differ considerably. Notably, our results show significant overlap with the distribution hotspots of threatened plants in China (including ferns, seed plants, cyanobacteria, and fungi) (Zhang and Ma 2008). Further analysis revealed that 74% of threatened medicinal gymnosperms have distribution ranges of less than 50 grids, and only one species exceeds 100 grids (Table S10), indicating that the threat status of medicinal gymnosperms is related to their distribution range, with narrowly distributed species more likely to face survival threats (Xie et al. 2021). However, not all threatened medicinal gymnosperms are narrowly distributed; for example, *Taxus wallichiana* is found in 178 grids (Table S10), which is relatively wide, but it has experienced sharp population declines and severe habitat destruction in recent decades due to its medicinal (paclitaxel extraction) and economic (timber) value (https://www.iucnredlist.org/). Therefore, human activities are also a major factor threatening medicinal gymnosperms (Corlett 2025).

Li et al. (2021) found that the species richness hotspots for all, threatened, and endemic gymnosperms in China were all in the Hengduan Mountains region. In this study, the species diversity hotspots for all and endemic Chinese medicinal gymnosperms are consistent with the results of Li et al. (2021), but the diversity hotspot for threatened medicinal gymnosperms is in northern Guangxi and the Wuyi Mountains. Future conservation efforts should focus on northern Guangxi. Furthermore, northern Guangxi is both a distribution hotspot for all medicinal gymnosperms and for threatened and nationally key protected medicinal gymnosperms; the Wuyi Mountains area also has rich threatened and nationally key protected medicinal gymnosperms (Figure 2). Such areas with multi‐dimensional conservation value should be prioritized.

### 4.3 Optimizing Conservation Strategies for Chinese Medicinal Gymnosperms

Establishing nature reserves is the most effective method for in situ biodiversity conservation, but how to maximize conservation benefits with limited resources remains a focus in academia (DeFries et al. 2005; Brooks et al. 2006; Watson et al. 2014). Identifying priority areas based solely on overall species richness is far from sufficient, as hotspots of high overall richness often contain assemblages of common and widely distributed species rather than rarer or more irreplaceable species, such as endemics, threatened species, or species significant for national economy, culture, research, and ecology. Protecting such species is crucial for reducing biodiversity loss (Reid 1998; Brooks et al. 2006; Carwardine et al. 2007; Lu et al. 2021). However, considering only species' irreplaceability also does not achieve optimal conservation outcomes; integrating both aspects can yield better results with less effort. This study offers two approaches to address these issues. First, applying the optimal algorithm to solve conservation planning problems. Previous studies have found that compared to other algorithms, the optimal algorithm often finds an optimal solution or a solution within a specified gap of the optimum, making it more suitable for practical conservation planning problems (Beyer et al. 2016). This study used the optimal algorithm to identify 22, 13, 17, and 12 grids as priority areas for all, endemic, threatened, and nationally key protected medicinal gymnosperms, respectively. Using the Dobson algorithm yielded 26, 15, 18, and 14 grids, respectively (Table S12). Under the premise of protecting the same number of species, the optimal algorithm resulted in fewer priority grids, achieving the goal of protecting more species with fewer resources. Furthermore, there are some unreasonable planning problems in the current protected area network in China, and these unreasonably set protected areas will cause a waste of national human and material resources for a long time (Fuller et al. 2010; Xu et al. 2019). Using the optimal algorithm can optimize the existing reserve network by replacing areas with low conservation contribution (Fuller et al. 2010). However, the core requirement for using the optimal algorithm is more precise and sufficient species geographical distribution information, necessitating increased investment in field surveys and the compilation of more detailed and accurate floras in the future. Particular attention should be paid to species with missing or scarce distribution records, such as *Cedrus deodara* . Second, establishing a conservation framework that integrates multi‐dimensional values. Conservation planning should integrate the multi‐dimensional values of species, including species richness and irreplaceability (Brooks et al. 2006; Carwardine et al. 2007). Most previous studies focused on a single type of value, rarely providing conservation suggestions combining multi‐dimensional values of species (Vega et al. 2006; Chi et al. 2017; Mehta et al. 2020; Xie et al. 2021). The conservation framework in this study comprehensively considers the richness of four dimensions: all medicinal gymnosperms, threatened medicinal gymnosperms, endemic medicinal gymnosperms, and nationally key protected medicinal gymnosperms, effectively compensating for the shortcomings of previous approaches relying on single indicators. Additionally, for medicinal gymnosperm species whose native habitats are severely damaged and populations are greatly threatened, *ex situ* conservation measures, such as relocation to botanical gardens or seed banks, are needed to maintain their survival or provide resources for future reintroduction into the wild (Li and Pritchard 2009; Zhou et al. 2021).

The identification of eight conservation gap grids (Figure 5; Table S13) provides new spatial evidence for protection shortfalls in the current reserve network. Notably, the gap identified in the Hengduan Mountains (grid 231, containing 37 species) indicates that even well‐studied biodiversity hotspots may still contain protection gaps when assessed using standardized spatial units. In addition, the gap at the Anhui–Hubei border (grid 391) highlights a priority area where threatened, endemic, and nationally protected medicinal gymnosperms co‐occur outside existing reserve networks.

The conservation prioritization framework developed in this study is readily transferable to other taxonomic groups, such as medicinal angiosperms or fungi, provided that several key steps and data requirements are satisfied. First, comprehensive species occurrence data must be compiled from surveys, literature, or herbarium databases and standardized at a consistent spatial resolution (e.g., county‐level records projected onto grid cells) to enable spatial distribution mapping. Second, species should be classified into relevant conservation dimensions—such as endemic status, threatened status based on the IUCN Red List, or nationally protected status—to allow multi‐dimensional prioritization. Third, species distribution data should be converted into a species‐by‐grid presence–absence matrix, which serves as the input for the conservation prioritization analysis. Once these data are prepared, the optimal algorithm can be applied to identify the minimum set of planning units that represent all species. Because the set‐cover framework is taxon‐agnostic, the same analytical procedure can be implemented for other taxonomic groups using the Julia package (Yang 2022).

The implementation of conservation actions in the identified priority grids may face important socio‐economic and governance challenges. Many of these grids are located in mountainous regions such as the Hengduan Mountains (grids 231, 303), the Wuyi Mountains, and the Qinling–Daba Mountains, where complex terrain and limited accessibility can complicate reserve establishment and management (Tang et al. 2006). In addition, several priority areas occur near provincial borders (e.g., grid 391 at the Anhui–Hubei border, grids 145 and 239 at the Guizhou–Guangxi border), requiring inter‐provincial coordination and potentially interacting with decentralized conservation governance structures in China (Xu et al. 2017). Such border regions may experience fragmented enforcement and differing provincial conservation priorities. Effective conservation will therefore require not only scientific prioritization but also mechanisms for cross‐jurisdictional collaboration, engagement with local communities, and consideration of livelihood needs in these often economically disadvantaged mountain regions. Future conservation planning should integrate stakeholder analysis and participatory approaches to address these implementation barriers.

It is important to acknowledge a key limitation of our conservation gap analysis: we considered only national nature reserves and national parks, excluding provincial and municipal protected areas. This is mainly because comprehensive and spatially explicit datasets for sub‐national reserves are not yet consistently available at the national scale. As a result, some priority grid cells identified as conservation gaps may already contain provincial or municipal protected areas, meaning that our estimates may overrepresent the extent of protection gaps. However, the magnitude of this potential effect cannot be quantified with the current dataset. Moreover, the conservation effectiveness of sub‐national reserves varies considerably, as many face challenges related to limited funding, staffing, and enforcement compared with national reserves (Xu et al. 2017). From a practical perspective, strengthening or upgrading existing sub‐national reserves within priority grids may represent a more feasible and cost‐effective strategy than establishing entirely new national reserves, consistent with China's recent efforts to optimize its protected‐area system (Xu et al. 2019). Future work should prioritize compiling comprehensive geospatial datasets of sub‐national protected areas to enable more accurate conservation gap analyses. In addition, emerging artificial intelligence (AI) tools may further enhance biodiversity monitoring and data integration, thereby supporting future conservation prioritization efforts (Ullah et al. 2025).

## 5 Conclusions

This study systematically analyzed the diversity of Chinese medicinal gymnosperms, revealed the geographical distribution patterns and hotspot areas of all, threatened, endemic, and nationally key protected medicinal gymnosperms, and constructed a comprehensive priority conservation framework integrating multi‐dimensional species values, considering species richness, threat status, endemism, and importance to national economy, culture, research, and ecology. By addressing the three research questions proposed in the Introduction—concerning spatial divergence among conservation categories, algorithmic efficiency, and protection gaps—this study provides a quantitative basis for prioritizing conservation actions for medicinal gymnosperms in China. By overlaying priority conservation areas with national nature reserve and national park layers, this study found gaps in the coverage of medicinal gymnosperms within the existing protection system. It is recommended that future conservation planning implement targeted measures gradually according to the levels of conservation gaps identified in this study. Based on our findings, we propose the following priority conservation actions:

1. *Prioritize formal protection of the Hengduan Mountains gap grid*. Grid 231 (Daocheng–Muli area, Sichuan Province) contains the highest richness among all gap grids (37 medicinal gymnosperm species) but currently lacks national‐level protection. Establishing a new nature reserve or upgrading existing provincial reserves in this area should be considered within upcoming conservation planning cycles.
2. *Establish cross‐provincial conservation collaboration for the Anhui–Hubei border gap*. Grid 391 (Jinzhai–Luotian area) harbors 22 species spanning threatened, endemic, and nationally protected categories. Because this priority area crosses provincial boundaries, coordinated conservation management between Anhui and Hubei provinces will be essential.
3. *Expand existing reserves to include adjacent priority grids*. Some priority grids located near existing protected areas—such as those in northern Yunnan and the Dalou Mountains—could be effectively protected by expanding current reserve boundaries, which may be more cost‐effective than creating entirely new protected areas.
4. *Regularly update conservation priorities using optimization‐based approaches*. As new distribution data become available, applying optimization frameworks such as the one used in this study can help conservation agencies reassess priority areas and improve the efficiency of resource allocation.

## Author Contributions

**Lisi Hai:** data curation (equal), formal analysis (equal), visualization (supporting), writing – original draft (lead). **Xinyi Wang:** data curation (lead), formal analysis (lead), visualization (lead), writing – original draft (supporting). **Yuchang Yang:** formal analysis (supporting), methodology (supporting). **Rui Shu:** formal analysis (supporting). **Yanqin Xu:** data curation (equal). **Guibing Lin:** data curation (equal). **Lihua Dong:** data curation (equal). **Xiaobo Zhang:** conceptualization (equal), supervision (equal), writing – review and editing (equal). **Zhangjian Shan:** conceptualization (equal), supervision (equal), writing – review and editing (equal).

## Funding

This work was funded by the Doctoral Research Foundation of Jiangxi University of Chinese Medicine (2023BSZR014), the Jiangxi Province Early‐Career Science and Technology Talent Support Program (20244 BCE52053), National Natural Science Foundation of China (Grant No. 82274052), and Science & Technology Fundamental Resources Investigation Program (Grant No. 2024FY100700).

## Ethics Statement

The authors have nothing to report.

## Consent

The authors have nothing to report.

## Conflicts of Interest

The authors declare no conflicts of interest.

## Supporting information


**Table S1:** The list of Chinese medicinal gymnosperms.

**Table S2:** The list of native Chinese medicinal gymnosperms.

**Table S3:** The list of endemic Chinese medicinal gymnosperms.

**Table S4:** The list of threatened medicinal gymnosperms in China.

**Table S5:** The list of national key protected medicinal gymnosperms in China.

**Table S6:** The county level occurrence data compiled for 148 Chinese medicinal gymnosperms.

**Table S7:** The grid‐cell occurrence data of Chinese medicinal gymnosperms for four categories used in this study.

**Table S8:** The classification of Chinese medicinal gymnosperms by medicinal used parts.

**Table S9:** The Species richness of Chinese medicinal gymnosperms in grid cells for four Categories.

**Table S10:** Number of Grid Cells with Occurrences of Four Categories of Medicinal Gymnosperms.

**Table S11:** The priority conservation grid cells for Chinese medicinal gymnosperms for four categories identified by the optimal method algorithm.

**Table S12:** The comparence of priority conservation areas between the Dobson and the optimal method algorithm.

**Table S13:** Prioritization of protection levels based on conservation gap grid cells.

## Data Availability

All data generated or analyzed during this study are included in this published article and its supporting information S1.
